# In Memoriam: Professor Huib Ovaa (1973-2020): A Uniquely Brilliant and Enthusiastic Scientist, a Pioneer in Chemical Biology and the Ubiquitin Field

**DOI:** 10.3389/fchem.2020.00627

**Published:** 2020-08-20

**Authors:** Monique P. C. Mulder, Benedikt M. Kessler

**Affiliations:** ^1^Department of Cell and Chemical Biology, Oncode Institute, Leiden University Medical Centre, Leiden, Netherlands; ^2^Nuffield Department of Medicine, University of Oxford, Oxford, United Kingdom

**Keywords:** obituary announcement, pioneer, chemical biology, dedication letter, ubiquitin 26S-proteasome system

A devastating loss of a colleague, mentor, and friend with broad impact on the ubiquitin and chemical biology field. Professor Huib Ovaa had the remarkable ability to integrate chemistry across a seemingly infinite array of biological disciplines, and did so with a contagious enthusiastic, bold, and fearless attitude.

**Figure:**
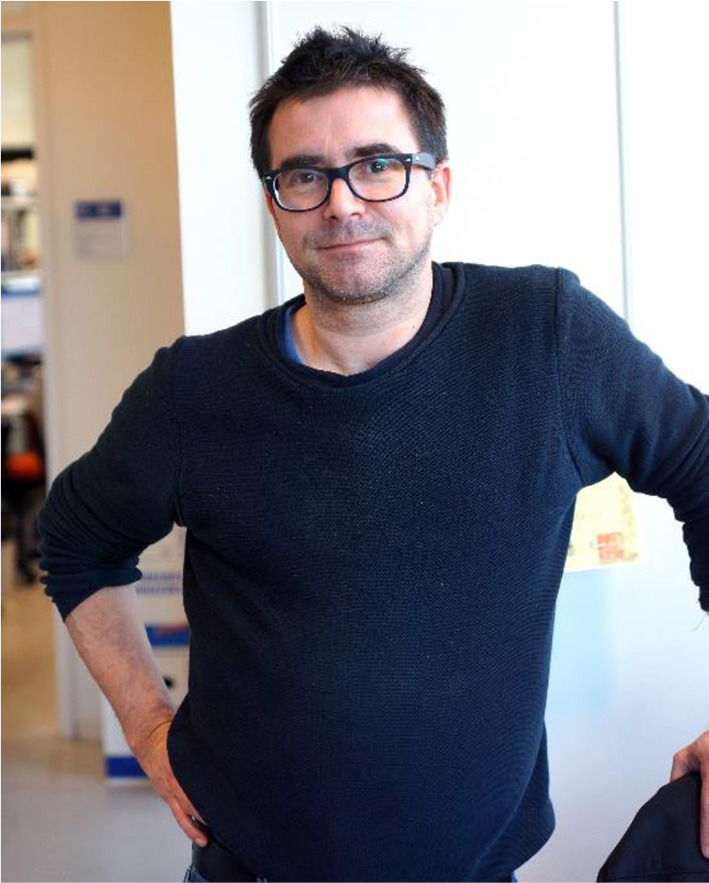
Professor Huib Ovaa (Photo by Duco van Dalen).

## Career

Huib was born on the 18th of December 1973 and spend most of his childhood in the westernmost and least populous province of the Netherlands, Zeeland. After his high school education (1987-1993, Middelburg, The Netherlands) he moved to Leiden to study chemistry at Leiden University (1993-1997). He commenced with his Ph.D. research at the same institution in 1997 in the laboratory of the late Prof. Jacques van Boom and spent part of his Ph.D. in the lab of Prof. Blechert at the TU Berlin, Germany. During this time, he was trained in the use of organometallic reactions on carbohydrate derived synthons to construct carbasugars and obtained his doctorate in 2001 with the distinction “Cum Laude.” After his Ph.D., Huib moved to the lab of Prof. Hidde Ploegh to perform his postdoctoral research at Harvard Medical School where he, as a fully trained synthetic organic chemist, became familiar with biochemistry and immunology and completed his academic formation.

In 2004 Huib returned to the Netherlands and was appointed Group Leader at the Netherlands Cancer Institute (NKI-AVL) in Amsterdam, where he started his own chemical biology lab. This lab was at the basis of many ubiquitin chemistries, proteasome technologies and MHC-exchange technologies used today and led to the establishment of the biotechnology spinoff company UbiQ. In 2011, Huib received the KNCV (Royal Dutch Chemistry Association) gold medal awarded to the best Dutch chemists under the age of 40 and was appointed Honorary Professor at the Leiden Institute of Chemistry (LIC), Leiden University in 2012. In 2016 Huib became Professor in Chemical Biology and at the same time his “Ovaa lab” moved to the Leiden University Medical Center (LUMC) to become part of the newly created department of Cell and Chemical Biology where he, until his death, supervised his research group and together with Prof. Jacques Neefjes headed the Chemical immunology groups.

## Major Contributions to the Ubiquitin and Chemical Biology Field

Huib's interest in chemical biology and eventually the ubiquitin proteasome system biology grew while he was conducting his postdoctoral fellowship in the laboratory of Prof. Hidde Ploegh at Harvard Medical School in Boston, USA. As being the only chemistry expert in a research environment of predominantly biologists and immunologists, he was exposed to many biological problems in antigen presentation in immune cells. This provided a perfect fertile ground to spark his interest in applying chemical tools to biological questions. Hidde Ploegh as his postdoctoral mentor encouraged that kind of thinking, in particular its application to immunology and antigen presentation related research. Subsequent discussions with colleagues with immunology expertise led to the realization that there was a need to better understand protein degradation, for antigen processing in the context of MHC class I, but also class II antigens, the latter through cross-presentation. In cells, protein degradation is predominantly mediated by ubiquitin conjugation to protein substrates. At that time, enzymes that recognize and process ubiquitin such as ubiquitin E3 ligases and deubiquitylating enzymes (DUBs) were sparsely characterized, perhaps with the exception of some prominent cases. To better understand ubiquitin-mediated protein degradation, creative thinking was required to face the challenges of generating tools by chemistry-based approaches. DUBs, cleaving ubiquitin chains, offered themselves as an attractive entry point for chemical tool development in forms of “molecular probes” through electrophilic moieties that could trap nucleophilic amino acid side chains of catalytic residues, such as cysteine. Originally, molecular principles borrowed from proteasome and cathepsin probes were transferred to the ubiquitin protein. Initially, the student project of Anna Borodovsky in the Ploegh lab, achieved this enzymatically via a reverse trypsin reaction to create ^125^I-labeled radioactive ubiquitin active site probes that were used to demonstrate functional interdependence between USP14 and the proteasome proteolytic activity (Borodovsky et al., 2001). With Huib's input, this concept was further developed into a panel of HA-tagged Ub probes with different chemical warheads, which differentially target cellular DUBs, leading to the discovery of ovarian tumor domain containing proteases (OTUs) as a novel subfamily of DUBs (Borodovsky et al., 2002). These studies yet again confirmed that Huib Ovaa had an extraordinary scientific talent and was able to link his remarkable chemical knowledge with relevant biological problems, in particular in the ubiquitin field.

After Huib's return to The Netherlands, he continued to make major contributions in this area. His unusual talent to think “out of the box” provided the framework for unconventional directions, occasionally in dispute with his peers, but provoking some astonishing discoveries.

Boris Rodenko, about the early days in the Ovaa lab: “When Huib started as a group leader at the NKI, I was lucky enough to join him as one of his first postdocs. The aptly minted ‘chemical biology' group at the NKI started with only a couple of people to conquer the world. Many of the NKI biologists looked at us with suspicious eyes. “We are not Pharma!” was a phrase often uttered by the NKI scientists in those days. Huib and the group would just laugh at this. Little did they know that we had a lot more to offer, but they would soon find out. With lots of energy Huib, and we in his team, started to develop the tools that have been making such an impact in the ubiquitin-proteasome and antigen presentation field ever since. And Huib had a big part not just in the design of the experiments, but also in their execution. In the afterhours and weekends, when the labs were quiet, he would pick a fume hood and started to brew all sorts of fluorescent reagents and probes to be used by the group or by collaborators. You would just pray to the heavens that he didn't pick your fume hood, as typically it would look like a bright pink fluorescent bomb had exploded there after Huib was done.”

For instance, under his directorship and together with his colleagues, the full synthesis of Ub/Ubl probes with remarkable yields was achieved via solid-phase peptide synthesis SPPS (El Oualid et al., 2010; Mulder et al., 2018). This was at odds with the status quo at that time on what solid-phase peptide synthesis technology can achieve, but his trick was to introduce di-amino acid Fmoc building blocks, thereby getting around challenging coupling steps.

**FIGURE 1:**
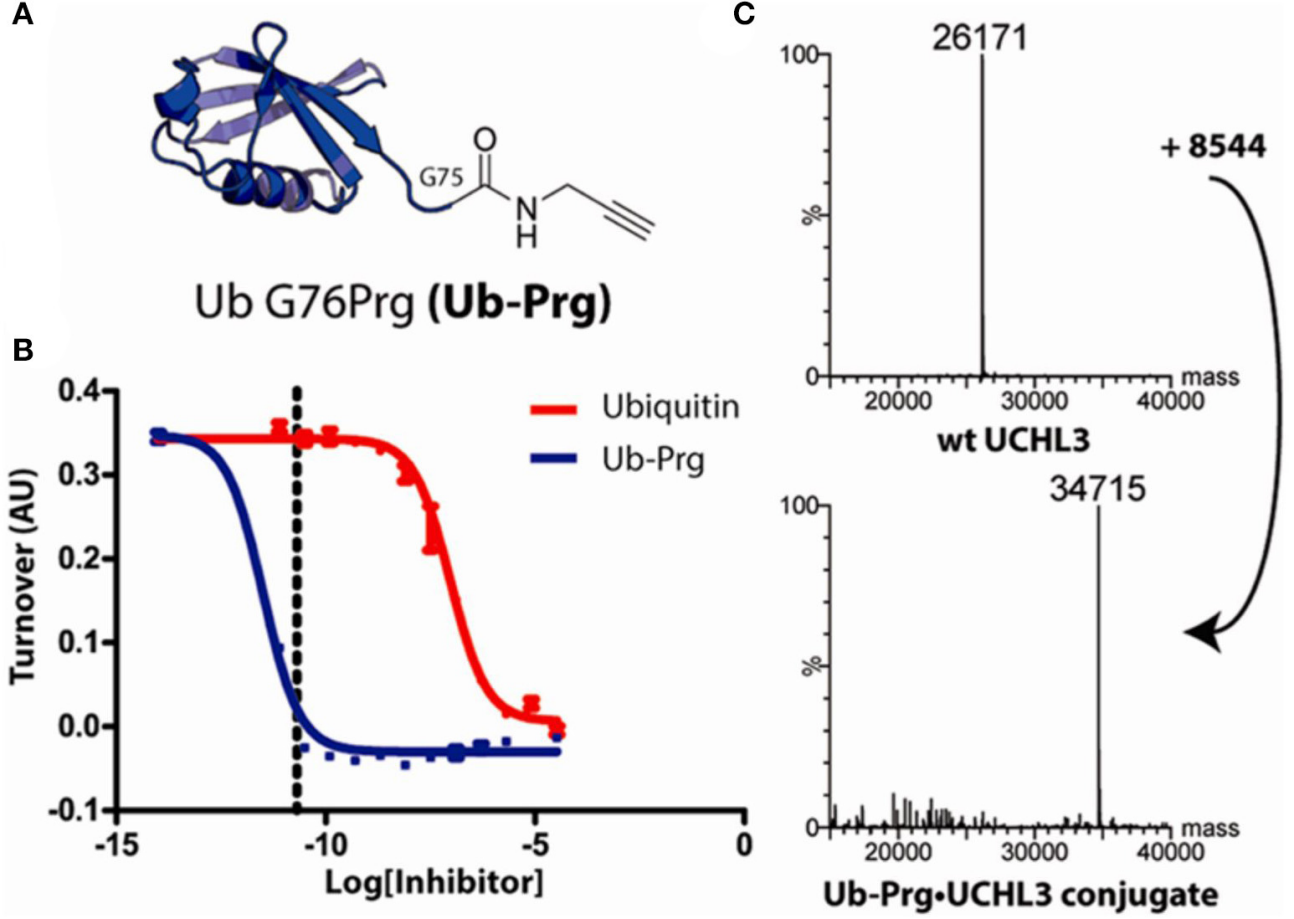
Ub-C-terminal alkyne reacts with UCH-L3 catalytic cysteine (Ekkebus et al., 2013) [With permission –Figure 1.]. **(A)** Structure of ubiquitin propargylamine Ub-PRG. **(B)** Fluorescence polarization-based substrate turnover assay measuring UCHL3 activity, showing Ub-Prg as 105 times more powerful an inhibitor than Ub. Dotted line represents UCHL3 concentration of (60 pM). **(C)** Mass spectra showing UCH-L3 ~ UbPRG adduct formation.

**FIGURE 2:**
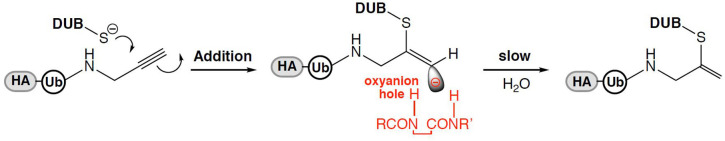
Proposed mechanism for Ub propargyl amine probes via a sterically controlled direct addition reaction with DUBs of the cysteine protease family [Abigail Schofield and Benedikt Kessler].

Moreover, in collaboration with David Komander, with whom he subsequently has made other landmark contributions to the ubiquitin field, he discovered that terminal alkynes, currently thought to be poor electrophiles and therefore “orthogonal” to chemical reactions in biological systems, can react with active-site cysteine nucleophiles in proteases (Figure 1). Ubiquitin active site probes carrying a propargyl warhead were shown to be compatible with thiol mod in DUBs (Ekkebus et al., 2013). This unexpected reaction involving electron rich alkynes is not yet completely understood, but perhaps is facilitated by the sterically correctly placed oxyanion hole in the catalytic center of proteases such as DUBs, leading to a direct addition reaction yielding a vinyl thioether as covalent adduct (Figure 2). Again, this was completely unexpected, at least to occur under physiological conditions, but also applies for other proteases (Arkona and Rademann, 2013; Mons et al., 2019).

**FIGURE 3:**
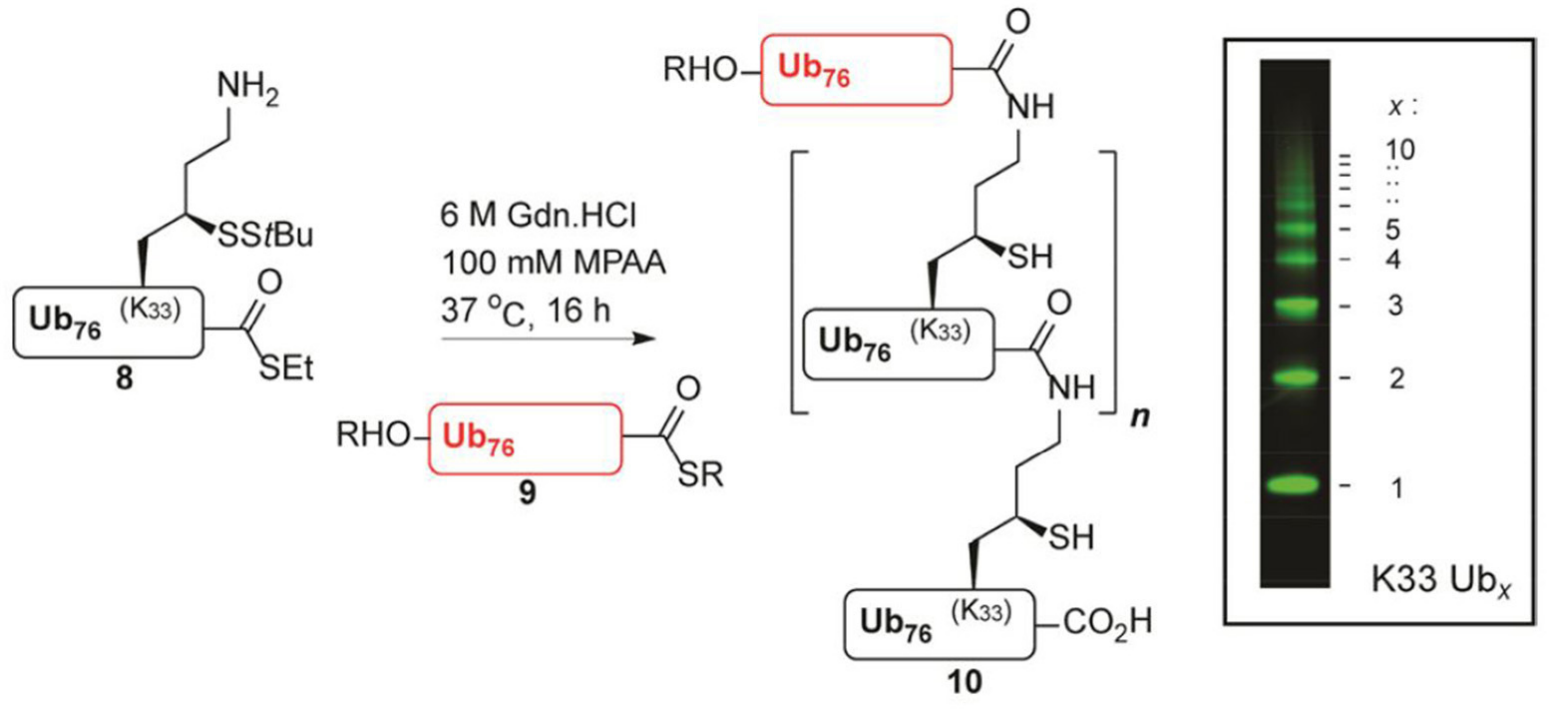
Polymerization reaction of K33 γ-thiolysine ubiquitin thioester (Van Der Heden Van Noort et al., 2017) [With permission—Scheme 3].

Another example is the remarkable elegance of oligo-Ub chain synthesis using a variation of thiolysine chemistry developed in Huib's laboratory (Van Der Heden Van Noort et al., 2017). This represents an optimized large scale and highly reproducible route to orthogonally protected γ-thiolysine and its use was demonstrated in the synthesis of bifunctional ubiquitin monomers (Figure 3). The edge of this approach is that these ubiquitin synthons are employed in polymerization reactions, giving access to synthetic poly-ubiquitin chains of defined linkage. In addition, compared to other efforts in synthesizing defined ubiquitin chains in the field, these are practically identical to their native counterparts, even leaving intact iso-peptide bonds after desulphurization.

Gerbrand van der Heden van Noort says: “Huib's way of mentoring was letting you run around the lab and do your own thing. Once in a while he would pop his head around the corner of the lab and ask if you wanted to have a look at your ongoing projects. If declined, he would disappear and put recent literature you might have missed with some unreadable notes on it on your office desk. Huib taught me to not worry about politics or peer pressure, ‘just be honest and focus on the science, the rest will fall into place later'. He was extremely proud on his lab, both on the personnel and facilities he had at his disposal. Having hard-core organic chemists, peptide chemists, protein chemists, biochemists, structural biologists, cell biologists and everything in between working side by side, he applauded every collaboration between his trainees and often encouraged the ‘lone wolfs' amongst us to seek and use each other's expertise.”

Ubiquitin based probes and defined Ub-chains have enabled many functional studies in collaboration with other experts in the ubiquitin field with whom Huib has had the chance to work productively, such as Ton Schumacher, David Komander, Titia Sixma, Ivan Dikic, Christopher Lima, Brenda Schulman, Hans-Peter Knobeloch, Jacques Neefjes, and others. The demand for high-quality Ub/Ubl tools in ubiquitin-related research has led to the foundation of the start-up company UbiQ.

**FIGURE 4:**
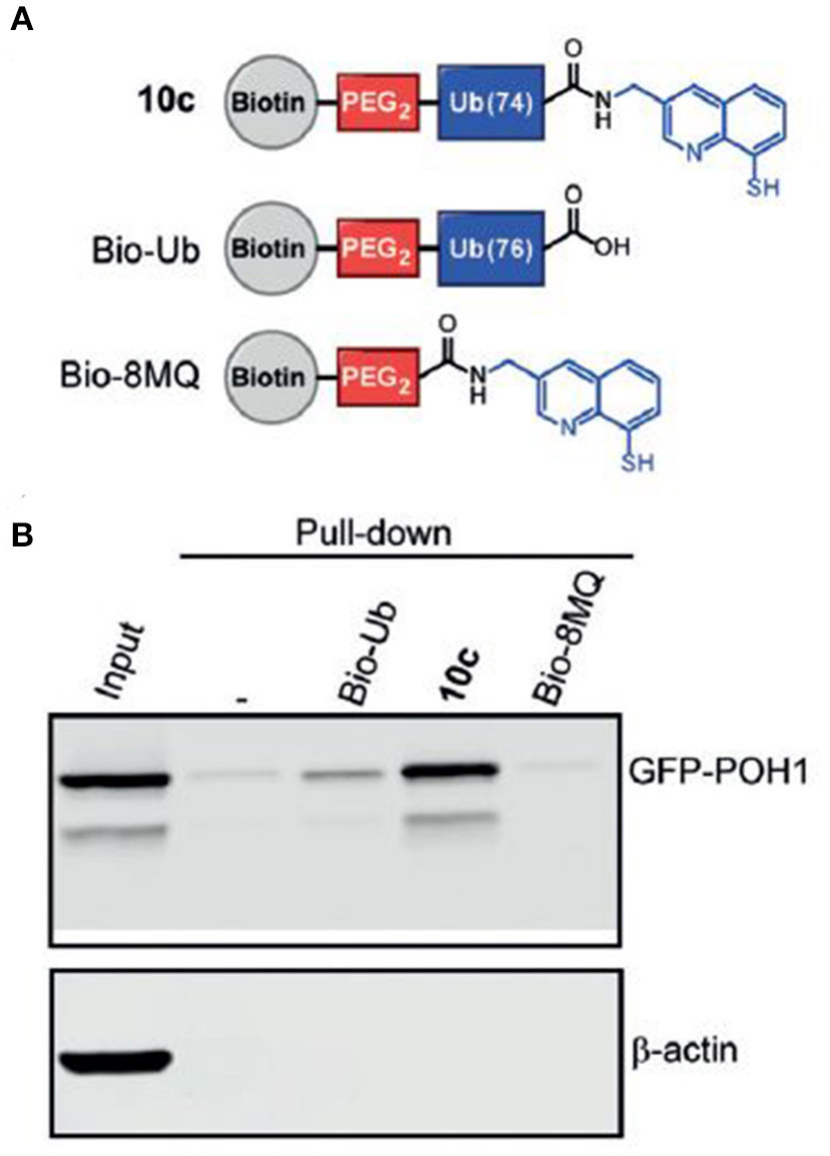
Novel metalloprotease probes (Hameed et al., 2019) [With permission—Figure 5A]. **(A)** Schematic representation of pull-down reagents used in the assay. **(B)** Western Blot analysis of pull-down from cell lysate of HeLa cells overexpressing GFP-POH1.

More recent developments initiated by Huib and his team included breakthroughs in challenging areas such as the first molecular probes for ubiquitin E3 ligases (Mulder et al., 2016) as well as probes that can target metalloprotease DUBs (Hameed et al., 2019). The latter, which provides access to an interesting subset of the DUB enzyme family, has been attempted previously for many years. It is, however, a reflection of Huib's remarkable efforts that made a change in this area by introducing an efficient zinc-chelator moiety at ubiquitin's C-terminus (Figure 4).

Additionally, his input into providing probes for studying DUBs at a cellular level helped to accelerate drug development targeting relevant DUBs in cancer, immunity, and neurodegeneration (Harrigan et al., 2018).

*Dharjath Shahul Hameed says: “Huib's lab has been the launching pad for many students. When I joined his lab as a Master- student intern, I was immediately given the freedom to work on so many ideas that would otherwise be generally shelved for a later day. When I got carried away with some ideas, he immediately reminded me to be practical and pragmatic. He was always up for challenges. There was a time when we faced strong headwinds in our metalloDUB probe project, and he gave us a big push by showing us how to combine chemistry and biology to solve one of the missing pieces of the ubiquitin puzzle. His approach is simple: a great idea executed in a simple way*.

When it comes to dealing with paperwork for foreign employees, he made sure we were not distracted by such bureaucracy. He always made sure we focus on research first and let the human resource management team worry about our visa and work permits. For him, science precedes bureaucracy, even in collaborations. For a foreigner and a student like me, he will remain as one of the best bosses anyone can wish for.”

**FIGURE 5:**
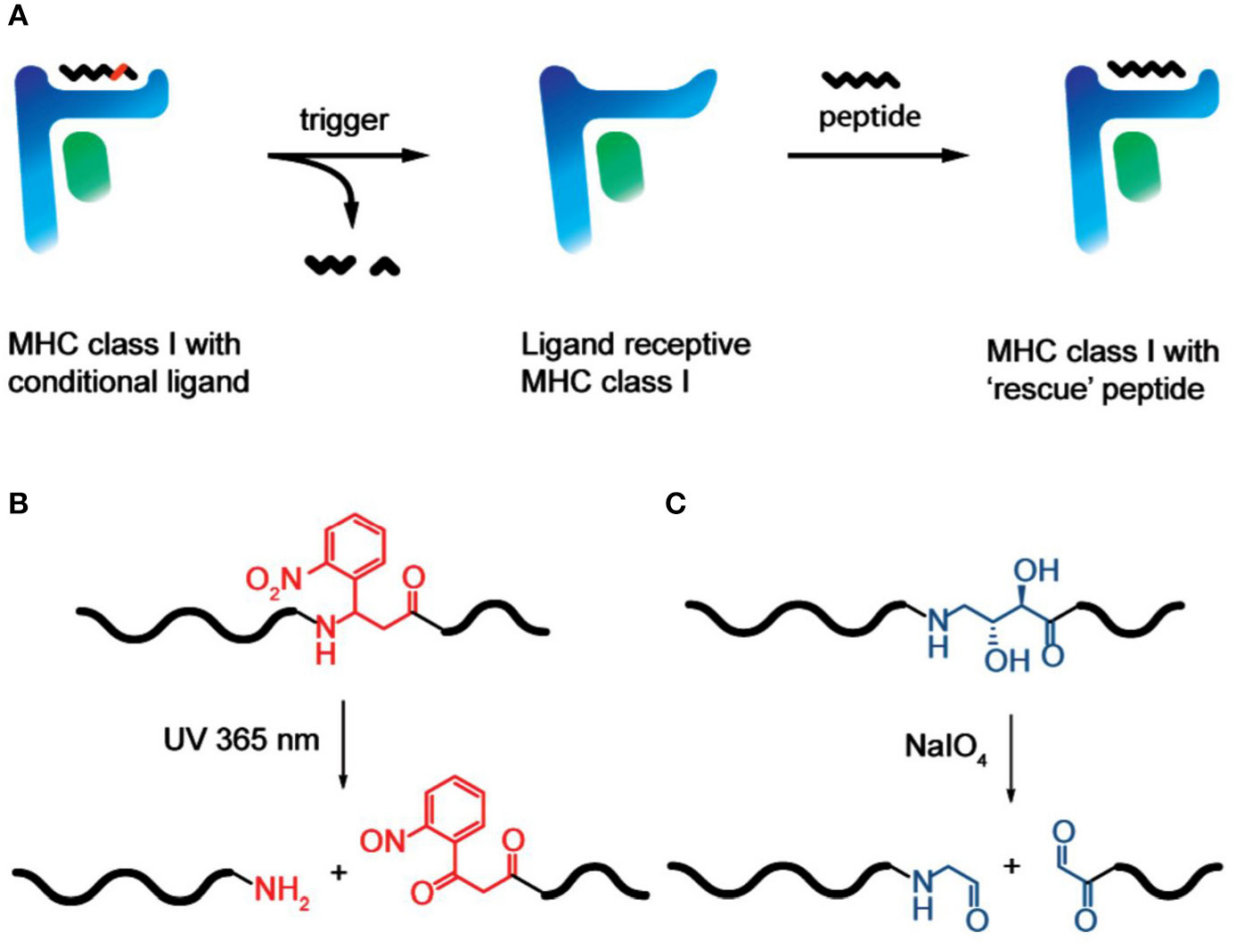
MHC class I peptide loading technology via periodate trigger (Rodenko et al., 2009). [With permission—Figures 1A–C]. **(A)** Conditional MHC class I is treated with a trigger, which cleaves the conditional peptide ligand to afford two fragments that no longer meet minimal affinity requirements and dissociate from the peptide binding groove. The resulting ligandreceptive MHC class I has a short half-life at 37 °C if not stabilized by the binding of a “rescue” ligand. **(B)** Photocleavage of a 2-nitrophenyl-containing conditional peptide ligand, triggered by 365 nm UV light. **(C)** Chemocleavage of a vicinal diol-containing conditional peptide ligand, triggered by the addition of sodium periodate.

Another unconventional contribution that Huib made, in collaboration with Ton Schumacher, to the field of immunology and antigen presentation was his clever design of MHC class I photoreactive peptides to enable efficient peptide loading (Toebes et al., 2006). The chemical “trick” used here was to introduce a diol-moiety into the antigenic peptide backbone that can be efficiently cleaved by sodium periodate (NaIO_4_), leading to the cleavage and removal of peptide remnants from the MHC class I peptide groove, making room for other peptides to bind, yielding MHC class I—peptide complexes of defined composition (Rodenko et al., 2009) (Figure 5). This elegant technical advance has provided an enormous boost to prepare MHC-I-peptide tools for the detection of circulating T-lymphocytes of a given antigen specificity, extremely relevant to study immune responses to cancer and infection. More recently, he and his colleagues expanded on this early work and developed an efficient method to generate many different MHC-I multimers in parallel using temperature-mediated peptide exchange (Luimstra et al., 2018).

Aysegul Sapmaz says: “I came to Huib's lab as a visiting Ph.D. student with a hardcore molecular biology background but a novice in chemistry. During that time, I was excited to use his magical tools for my Ph.D. study. Even though it was a short stint, I already realized that I would love to continue working with him. When I asked for a postdoc position in his lab, he did not hesitate and immediately said “Yes”. From the moment I started in his lab, he always encouraged me to believe in my gut feelings and let me work freely on my own projects even if there was very little overlap with chemistry. He also encouraged me to provide my biological expertise in other chemistry-based projects going on in the lab. This has resulted in several publications in the field of applied chemistry with many more on the way. Huib always supervised the people in his lab by trusting and encouraging them to think out of the box and to do the impossible. When you had interesting results, he got super excited which put his signature big smile on his face. He was a great mentor who always supported his team during difficult times both in their scientific and personal life. He was always proud of his lab that he meticulously built with trust, freedom, and encouragement.”

His lab, jointly run with Prof. Jacques Neefjes, has recently expanded as part of the Chemical Immunology Unit at LUMC, a tribute to their scientific success. Huib was an extraordinary scientist who fearlessly extended his research into a very broad spectrum of the scientific field, as exemplified by one of his more recent publications (Sapmaz et al., 2019). In collaboration with Prof. Jacques Neefjes, they revealed the function of a previously unidentified DUB, USP32, using molecular biology and biochemistry approaches. His most recent research was geared toward the development of small molecule inhibitor molecules against DUBs as tool compounds and precursors of drug development (Geurink et al., 2019).

Huib has left us way too early as a colleague and exceptional scientist. We could have expected so much more coming from him—he will be remembered as a bright star in the sky of ubiquitin & chemical biology.

## Author Contributions

MM and BK wrote and edited the manuscript and approved it for publication.

## Conflict of Interest

The authors declare that the research was conducted in the absence of any commercial or financial relationships that could be construed as a potential conflict of interest.
